# Antioxidant capacity and fatty acids characterization of heat treated cow and buffalo milk

**DOI:** 10.1186/s12944-017-0553-z

**Published:** 2017-08-24

**Authors:** Imran Taj Khan, Muhammad Nadeem, Muhammad Imran, Muhammad Ayaz, Muhammad Ajmal, Muhammad Yaqoob Ellahi, Anjum Khalique

**Affiliations:** 1 Department of Dairy Technology, University of Veterinary and Animal Sciences, Lahore, Punjab Pakistan; 2 Institute of Home and Food Sciences, Faculty of Science and Technology, Government College University, Faisalabad, Punjab Pakistan; 3 Army College of Veterinary Sciences, Sargodha, Punjab Pakistan; 4 Department of Animal Nutrition, University of Veterinary and Animal Sciences, Lahore, Punjab Pakistan

**Keywords:** Heat treatment, Cow milk, Buffalo milk, Antioxidant capacity, Boiling, Pasteurization

## Abstract

**Background:**

Antioxidant capacity of milk is largely due to vitamins A, E, carotenoids, zinc, selenium, superoxide dismutase, catalase, glutathione peroxidase and enzyme systems. Cow milk has antioxidant capacity while the antioxidant capacity of buffalo milk has been studied in a limited way. The information regarding the effect of pasteurization and boiling on antioxidant capacity of cow and buffalo milk is also scared.

**Methods:**

Cow and buffalo milk was exposed to two different heat treatments i.e. 65 °C for 30 min and boiling for 1 min. After heat treatments, milk samples were cooled down to 4 °C packaged in transparent 250 ml polyethylene PET bottles and stored at 4 °C for 6 days. Milk composition, total flavonoid content, total antioxidant capacity, reducing power, DPPH free radical scavenging activity, antioxidant activity in linoleic acid, vitamin C, A, E, selenium, Zinc, fatty acid profile, peroxide value and sensory characteristics were studied in raw, pasteurized and boiled cow and buffalo milk at 0, 3 and 6 days of storage period.

**Results:**

Total antioxidant capacity (TAC) of raw, pasteurized and boiled milk for cow (42.1, 41.3 and 40.7%) and buffalo (58.4, 57.6 and 56.5%) samples was found, respectively. Reducing power (RP) of raw cow and buffalo milk was 6.74 and 13.7 while pasteurization and boiling did not showed significant effect on RP of both cow and buffalo milk. DPPH activity of raw, pasteurized and boiled milk for cow (24.3, 23.8 and 23.6%) and buffalo (31.8, 31.5 and 30.4%) samples was noted, respectively. Storage period up to 3 days was non-significant while DPPH assay after 6 days of storage period indicated significant decline in antioxidant activity of milk samples. Antioxidant activity in linoleic acid (AALA) of buffalo and cow milk were recorded 11.7 and 17.4%, respectively. Pasteurization and boiling did not showed any impact on antioxidant capacity of cow and buffalo milk. The Loss of vitamin C in pasteurization (40 and 42%) and boiling (82 and 61%) of cow and buffalo milk was recorded, respectively. Concentration of vitamin A and E in pasteurized cow and buffalo milk was not significantly different from raw milk samples of cow and buffalo. Concentration of selenium and zinc was not influenced by the heat treatment in both cow and buffalo milk samples. After 3 days of refrigerated storage, antioxidant capacity of both cow and buffalo milk decreased. Concentrations of short-chain and medium-chain fatty acids increased in pasteurized and boiled cow and buffalo milk, while long-chain fatty acids decreased in pasteurized and boiled cow and buffalo milk, with no effect on colour and flavor score. Peroxide value of pasteurized and boiled cow and buffalo milk was not influenced by the storage up to 3 days.

**Conclusions:**

These results suggest that buffalo milk had a higher antioxidant capacity than cow milk and pasteurized milk should be consumed within 3 days of refrigerated storage for better antioxidant perspectives.

## Background

Buffalo milk is contributing 12% of the total milk production in the world. About 80% of total buffalo milk is produced in India and Pakistan [1]. In subcontinent, buffalo milk is preferred over cow milk due to white color, higher fat, protein, total solids contents and creamy taste [2]. Buffalo milk is highly suitable for the manufacturing of wide range of value added dairy products, such as, yoghurt, mozzarella and cheddar cheese [3]. Buffalo milk is healthier than cow milk in terms of lower concentration of cholesterol and higher magnitude of unsaturated fatty acids [4]. Fat content of buffalo and cow milk ranges from 6 to 7% and 3.5–4.5%, respectively [5]. Protein content of buffalo and cow milk is 3.8–4% and 3.2–3.3%, respectively while ash content of buffalo and cow milk is 0.82% and 0.72%, respectively. The viscosity of buffalo milk is also greater than cow milk [6]. In addition to the normal nutritional perspectives, certain milk constituents have functional value. Antioxidants are chemical substances than scavenge/neutralize the free radicals and foods should contain enough concentration of antioxidants to prevent oxidative stresses. Uninterrupted reactive oxygen species can lead to diabetes, atherosclerosis, accelerated ageing, breakdown of DNA and several essential biochemical compounds [7]. Increased incidences of metabolic diseases have led the consumers to make healthy choices of foods and demand for functional foods is mounting across the world. Changing life styles have led the food industry and researchers to develop functional foods and determine the functional value of traditional foods. Increased knowledge in free radical biology has led the consumer to consume functional foods containing natural antioxidants. Casein, whey, sulphur containing amino acids, selenium, zinc, catalase, glutathione peroxidase, superoxide dismutase, vitamin E, C and beta-carotene has antioxidant activity in milk [8]. Concentration of vitamin E in buffalo and cow milk is 5.5 and 2.1 mg/ 100 ml, respectively while the amount of vitamin C in buffalo and cow milk is 3.66 and 0.94 mg/100 ml, respectively [9]. Buffalo milk has higher magnitude of sulfur containing amino acids, selenium and zinc as compared to cow milk [10]. Concentration of beta-carotene in cow milk is more than buffalo milk. Due to the difference in the concentration of antioxidant substances, buffalo and cow milks may have different antioxidant capacity. Antioxidant capacity of few fermented dairy products is reported in literature. Antioxidant capacity of goat milk based kefir was investigated by 2-Diphenyl-1-picrylhydrazyl assays and antioxidant capacity of kefir was more than native milk [11]. Antioxidant capacity of probiotic yoghurt was studied in cow, goat and camel milk using *Pediococcus pentosaceus* and it was observed that fermentation improved the antioxidant capacity of probiotic yoghurts [12]. Pasteurization is one of the most commonly used technique for the processing of fluid milk, in Pakistan, about 2–3% milk is pasteurized whereas, rest of the milk is boiled at homes for household applications. Effect of pasteurization and boiling on antioxidant characteristics of cow and buffalo milk should be studied for improved consumption patterns.

During pasteurization, milk is exposed to a certain heat treatment for a specific period of time and this time and temperature combination may have impact on antioxidant characteristics of milk. Similarly, storage time may also affect the antioxidant activity of milk and dairy products. Different heat treatments are performed to kill the pathogens in milk. Chemical composition of cheddar cheese is considerably different from parent milk [13]. Changes in antioxidant activity of pasteurized milk and cheddar cheese during pasteurization and ripening period are not previously discussed. Production of bioactive peptides during the ripening of cheddar cheese increased the antioxidant capacity of cheese [14]. However, effect of heat treatment and storage period on antioxidant characteristics of pasteurized milk from cow and buffalo milk is not previously investigated. Therefore, functional value of buffalo milk may be different from cow milk. Buffalo milk is widely used for the manufacturing of large number of value added dairy products and literature review evidenced that cow milk has antioxidant capacity, whereas, antioxidant capacity of buffalo milk is not previously studied. Detailed investigation on antioxidant perspectives of cow milk is also required for the better understanding of functional value of world’s largest source of milk. In such a situation, functional value of traditional foods should be discovered. This study was planned to determine the antioxidant characteristics of cow and buffalo milk on the basis of some chemical characteristics.

## Methods

### Raw materials

Cow and Buffalo milk was obtained from Dairy Animals Training and Research Centre, University of Veterinary and Animal Sciences Lahore, Pakistan. All the chemicals used in this study were GC-grade and obtained from Sigma Chemical Co. (St. Louis, MO, USA).

### Experimental plan

Cow and buffalo milk was exposed to two different heat treatments i.e. 65 °C for 30 min and boiling for 1 min. After heat treatments, milk samples were cooled down to 4 °C packaged in 250 ml polyethylene PET bottles and stored at 4 °C for 9 days.

Following antioxidant characteristics of heated treated milk were determined at 0, 3, and 6 days of storage period.

### Chemical composition of cow and buffalo milk

Milk composition of raw and heat treated cow and buffalo milk was determined on lactoscan (Julie Z7-Slovakia).

### Total flavonoid content

Total flavonoid content in milk samples were determined by AlCl_3_ spectrophotometric assay using Rutin as standard. In a test tube, milk sample (0.1 ml) was mixed with 0.2 ml (5% NaNO_3_), after 5 min 0.2 ml AlCl_3_ and 1 ml NaOH (1 Molar) was added, contents were incubated for 15 min at room temperature. Absorbance of the samples and standards was measured at 510 nm on a double bean spectrophotometer (Shimadzu, Japan). Total flavonoid content was calculated from the calibration curve and reported as mg RE/g [15].

### Total antioxidant activity

Total antioxidant activity in milk samples was determined according to the method of Nabasree and Bratati [16]. Sample 0.3 ml was mixed with 3 ml each of 0.6 M sulfuric acid, 28 mM sodium phosphate and 4 mM ammonium molybdate). Test tubes were incubated at 85 °C for 90 min, absorbance was recorded at 695 nm in visible region of spectrum using reagent blank and ascorbic acid solution as standard. Total antioxidant activity was reported as mg Ascorbic Acid Equivalent per gram.

### Reducing power

Reducing power of the milk samples was determined by following the method of Adesegun et al. [17] by mixing 2.5 ml sample with 2.5 ml potassium ferricyanide (1%), followed by incubation at 50 °C for 20 min. After incubation, 2.5 ml trichloroacetic acid solution (10%) was added and centrifuged 1000 × g, 10 min). Supernatant 2.5 ml was mixed with same volume of deionized water and 0.5 ml ferric chloride (0.1%) was added. Absorbance was recorded at 700 nm on a double beam spectrophotometer.

### 1,1-diphenyl-2-picrylhydrazyl (DPPH) free radical scavenging activity

DPPH free radical scavenging activity was determined by the method [17]. 1 ml milk samples were added with 1 ml of methanol solution of DPPH (1 mM), vortexed at 2200 rpm for 1 min and incubated at room temperature for 20 min, absorbance was recorded at 517 nm on a double bean spectrophotometer and DPPH free radical scavenging activity was reported in percentage.

### Antioxidant activity in linoleic acid system

Antioxidant activity of milk samples in linoleic acid system was determined by the method prescribed by Osawa and Namiki [18]. Milk sample 0.5 ml was mixed with 0.13 ml linoleic acid solution in 99.8% ethanol and 10 ml (0.2 M) sodium phosphate buffer, volume of mixture was diluted to 25 ml. Solutions were incubated at 40 °C and degree of oxidation was assessed by the thiocyanate method; 10 ml ethanol (75%), 0.2 ml ammonium thiocyanate, 10 ml ethanol (75%) and 0.2 ml ferrous chloride (20 mM in 3.5% hydrochloric acid). After mixing for 3 min, absorbance was measured at 500 nm on a double bean spectrophotometer and BHT was also used as positive control [19].

### Determination of vitamin E

Fat from milk was extracted by the standard method [20]. Concentration of Vitamin E in milk samples was determined on HPLC (Miford, MA; 715 Ultra WISP injector; 25 cm × 4.6 mm diameter 5-μm Supelcosil LC-Si; Supelco, Bellefonte, PA). 200 μg milk fat was mixed with 2 ml n-hexane (HPLC grade). The contents were vortexed at 1500 rpm for 25 s and injected into HPLC. Mobile was comprised of 0.5% ethyl acetate and 0.5% acetic acid in hexane with flow rate adjusted at 1.5 ml/min. Vitamin E was expressed as μg/g [21].

### Determination of vitamin A

For the determination of vitamin A in milk, 20 ml milk sample was added with 5 ml ammonia (25%) and 20 ml ethanol (96%). Supernatant was extracted and added with 0.0025% BHT. Solvent was evaporated at 35 °C on a rotary evaporator. Saponification was performed with 30 ml potassium hydroxide (5% in ethanol) followed by the extraction with *n*-hexane. Solvent was evaporated on rotary evaporator and 20 μl injected into HPLC equipped with LC-20 AT pumping system, waters 990 detector and Spherisorb RP-18 column (Shimadzu). Retinyl palmitate was used as standard in various concentrations. Mobile phase was comprised of acetonitril-methanol 85:15 in isocratic system [22].

### Determination of vitamin C

Vitamin C was determined by HPLC and for this purpose 300 μl milk samples were mixed with 300 μl 0.56% metaphosphoric acid, followed by centrifugation at 10 °C for 10 min at 3000 × g. measurement was performed at 254 nm using ascorbic acid as standard. Quantification of vitamin C was performed by calibration curve [23].

### Fatty acid profile

Fatty acids profile was determined as fatty acid methyl esters developed from 300-μL melted and well mixed sample. The sample was taken into 11-mL screw capped test tube, dissolved in 3-mL isooctane and 2-mL 0.5 N sodium methoxide was added. The samples were vortexed at higher speed for exactly 3-min and allowed to separate for 5 min. The supernatant was injected by auto sampler into Gas Chromatograph 7890-A Agilent, fitted with a methyl lignoserate-coated (film thickness 0.25 l m), SP-2330 (SUP ELCO Inc. Supelco Park Bellefonte, PA 16823–0048, USA) polar capillary column (30 m × 0.32 mm). Fatty acids were identified and quantified by FAME 37 internal standards [24, 25].

### Peroxide value

Peroxide value in raw, pasteurized and boiled cow and buffalo milk was determined at 0, 3 and 6 days of storage period according to the standard method [26].

### Determination of zinc and selenium

Selenium and Zinc were determined by the standard methods [27].

### Statistical analysis

Every sample was analyzed three times and the data were expressed as Mean ± SD (*n* = 3 × 3; ±SD *n* = 3 × 3). Data was analyzed by using two-way analysis of variance techniques to find out the effect of treatment and storage by using SAS 9.1 (Statistical Analysis Software) software. *P*-value of 0.05 and 0.01 was used to denote the significant and highly significant difference.

## Results and discussion

### Chemical composition of raw, pasteurized and boiled milk

Pasteurization and boiling did not have any effect on fat, protein, lactose, ash, solids not fat and total solids content (Table 1). Results of current investigation are not different from the earlier studies [10].

**Table 1:** Chemical composition of raw, pasteurized and boiled cow and buffalo milk

Parameter	Cow Milk			Buffalo Milk		
	Raw Milk	Pasteurized	Boil	Raw Milk	Pasteurized	Boil
Fat Content%	4.17 ± 0.13^b^	4.14 ± 0.05^b^	4.21 ± 0.11^b^	6.45 ± 0.16^a^	6.42 ± 0.08^a^	6.53 ± 0.07^a^
Protein%	3.22 ± 0.09^b^	3.19 ± 0.03^b^	3.26 ± 0.02^b^	3.82 ± 0.14^a^	3.80 ± 0.05^a^	3.88 ± 0.12^a^
Lactose%	4.54 ± 0.19^b^	4.52 ± 0.23^b^	4.61 ± 0.17^b^	4.85 ± 0.26^a^	4.87 ± 0.12^a^	4.94 ± 0.25^a^
Ash%	0.73 ± 0.04^b^	0.72 ± 0.02^b^	0.73 ± 0.05^b^	0.83 ± 0.01^a^	0.82 ± 0.07^a^	0.84 ± 0.06^a^
SNF%	8.65 ± 0.28^a^	8.58 ± 0.09^a^	8.75 ± 0.07^a^	9.65 ± 0.21^a^	9.61 ± 0.15^a^	9.81 ± 0.32^a^
Total Solids%	12.7 ± 0.34^b^	12.6 ± 0.28^b^	12.9 ± 0.30^b^	16.21 ± 0.43^a^	16.05 ± 0.24^a^	16.29 ± 0.18^a^

Within a row, means denoted by a different letter are statistically different (*P* < 0.05)

*SNF* Solids Not Fat

### Antioxidant characteristics of raw, pasteurized and boiled cow and buffalo milk

#### Total flavonoid content (TFC)

TFC of raw, pasteurized and boiled cow and buffalo milk are given in Table 2. Total flavonoid contents were expressed in Rutin Equivalent (mg/ml). TFC in buffalo milk was greater than cow milk. TFC of cow and buffalo milk were not influenced by pasteurization and boiling operations. Results of earlier investigation have shown that TFC of harshly processed foods was less than initial value [28]. Total flavonoid content was also influenced by the storage period while storage period up to 3 days was non-significant. The concentration of total flavonoids in raw, pasteurized and boiled cow and buffalo milk decreased in 6 days old milk. Flavonoids are secreted in milk from fodder and feed and furthermore magnitude depends upon the concentration of flavonoids in feed stuff, therefore, their concentration may vary, as they are feed derived biochemical compounds. It may be assumed that they are usually present in milk, however, this aspect requires detailed investigation with respect to feed, breed and seasonal effects. Antioxidant properties of flavonoids are scientifically established [29]. Little is known regarding the existing of flavonoids in cow and buffalo milk.

**Table 2:** Antioxidant characteristics of raw, pasteurized and boiled cow and buffalo milk

Parameter	Days	Cow Milk			Buffalo Milk		
		Raw Milk	Pasteurized	Boil	Raw Milk	Pasteurize	Boil
TFC Rutin Equivalent mg/ml	0	1.89 ± 0.04^c^	1.85 ± 0.05^c^	1.82 ± 0.12^c^	3.72 ± 0.06^a^	3.71 ± 0.14^a^	3.68 ± 0.07^a^
	3	1.87 ± 0.09^c^	1.84 ± 0.11^c^	1.81 ± 0.17^c^	3.68 ± 0.02^a^	3.65 ± 0.10^a^	3.63 ± 0.03^a^
	6	1.45 ± 0.03^d^	1.42 ± 0.02^d^	1.39 ± 0.13^d^	3.41 ± 0.07^b^	3.43 ± 0.18^b^	3.37 ± 0.02^b^
TAC %	0	42.1 ± 1.58^c^	41.3 ± 1.15^c^	40.7 ± 0.94^c^	58.4 ± 1.81^a^	57.6 ± 1.47^a^	56.5 ± 1.62^a^
	3	41.6 ± 0.87^c^	39.9 ± 1.61^c^	39.8 ± 1.16^c^	56.8 ± 1.21^a^	56.2 ± 1.66^a^	55.7 ± 1.84^a^
	6	37.5 ± 1.28^d^	38.4 ± 0.79^d^	37.5 ± 1.65^d^	52.7 ± 1.37^b^	49.8 ± 1.28^b^	47.4 ± 0.73^b^
RP	0	6.74 ± 0.53^c^	6.63 ± 0.71^c^	6.55 ± 0.44^c^	13.7 ± 1.19^a^	13.5 ± 0.93^a^	13.4 ± 0.55^a^
	3	6.51 ± 0.38^c^	6.14 ± 0.35^c^	5.87 ± 0.62^c^	12.9 ± 0.56^a^	13.3 ± 0.^72a^	12.6 ± 0.55^a^
	6	5.3 ± 0.62^d^	4.9 ± 0.22^d^	4.77 ± 0.35^d^	10.3 ± 0.43^b^	11.6 ± 0.31^b^	10.8 ± 1.26^b^
DPPH %	0	24.3 ± 0.49^d^	23.8 ± 1.10^c^	23.6 ± 0.58^c^	31.8 ± 1.77^a^	31.5 ± 0.67^a^	30.4 ± 0.94^a^
	3	23.7 ± 0.66^d^	23.1 ± 1.53^c^	22.7 ± 1.05^c^	30.6 ± 1.49^a^	29.7 ± 0.52^a^	29.5 ± 0.45^a^
	6	21.6 ± 1.23^e^	19.4 ± 0.91^e^	16.5 ± 0.27^e^	28.6 ± 0.84^b^	27.4 ± 1.17^b^	26.7 ± 1.22^b^
AA in LA %	0	11.7 ± 0.39^c^	11.3 ± 0.37^c^	11.1 ± 0.44^c^	17.4 ± 0.62^a^	17.1 ± 1.13^a^	16.8 ± 0.98^a^
	3	11.2 ± 0.44^c^	10.7 ± 0.53^c^	9.11 ± 0.19^c^	16.9 ± 0.33^a^	13.7 ± 0.70^a^	11.8 ± 0.62^a^
	6	8.55 ± 0.76^d^	8.76 ± 0.27^d^	8.73 ± 0.38^d^	15.8 ± 1.37^b^	11.2 ± 0.91^b^	10.4 ± 0.36^b^

Within the rows and columns of a parameter, means denoted by a different letter are statistically different (*P* < 0.05)

*TFC* Total Flavonoid Content (mg Rutin/g)

*TAC* Total Antioxidant Capacity

*RP* Reducing Power

DPPH

*AA in LA* Antioxidant Activity in Linoleic Acid

*H*_*2*_ *O*_*2*_ *FRSA* Hydrogen Peroxide Free Radical Scavenging Activity

#### Total antioxidant capacity of cow and buffalo milk (TAC)

TAC refers to the antioxidant status of biochemical compounds and measures the antioxidant response to counter the free radicals produced. TAC can be used as a novel marker for the assessment of oxidative stress [30]. TAC of buffalo milk was higher than cow milk, buffalo milk has higher concentration of vitamin C, E, selenium, zinc, tyrosine, cysteine, antioxidant activity of these chemical constituents is scientifically established. Catalase also possesses the antioxidant activity and catalase activity is 2–4 times higher in buffalo milk as compared to cow milk. This might be the justification for higher total antioxidant capacity of buffalo milk than cow milk. Pasteurization and boiling did not have any significant effect on TAC of cow and buffalo milk (Table 2). TAC of raw, pasteurized and boiled cow milk was 42.1, 41.3 and 40.7%, respectively. TAC of raw, pasteurized and boiled buffalo milk was 58.4, 57.6 and 56.5%, respectively. Zulueta et al. [31] stated that oxygen radical absorbance capacity of ultra high temperature treated milk and pasteurized milk was 13.17 and 13.93 μM trolox equivalents (*p* > 0.05), respectively. Total antioxidant capacity of raw, pasteurized, boiled, cow and buffalo milk remain unchanged during the storage period of 3 days, however, total antioxidant capacity of 6 days old raw, pasteurized and boiled cow and buffalo milk was significantly less than zero day. Jung et al. [32] mentioned that antioxidant capacity of the yoghurt fortified with red Ginseng extract decreased during the refrigeration storage. Jeong et al. [33] studied the impact of heat treatment on antioxidant capacity of citrus peels. Samples were exposed to 50, 100 and 150 °C for 10, 20, 30, 40, 50 and 60 min. Antioxidant activity of citrus peel increased as the temperature and time increased.

#### Reducing power (RP)

RP of raw cow and buffalo milk was 6.74 and 13.7, respectively while pasteurization and boiling did not have significant effect on RP of both cow and buffalo milk. After pasteurization, RP of cow and buffalo milk decreased by 1.63% and 1.45%, respectively from the raw milk samples. After boiling, RP of cow and buffalo milk decreased by 2.81% and 2.19%, respectively from the raw milk samples. Greater reducing in raw, pasteurized and boiled buffalo milk may be attributed to the existence of higher concentration of antioxidant substances. Antioxidant characterization of buffalo milk revealed that it is healthier than cow milk. Reactive oxygen species can lead to the degeneration of cell membrane, protein mutilation, DNA mutation, cancers and cardiovascular diseases [34]. Recent dietary guidelines suggest including natural antioxidant in the diet to avoid oxidative stress. In this regard, polyphenols such as flavonoids and phenolic acids have been found to have antioxidant properties [35]. Antioxidant capacity of milk and some fermented dairy products is described in literature. Antioxidant systems of milk can efficiently inhibit superoxide radicals, hydroxyl radicals and peroxide radicals (19, from antioxidant review paper). Ruiz de Gordoa et al. [36] studied the effect of grazing on antioxidant capacity of sheep milk, grazing enhanced the antioxidant capacity of milk. Roy and Deepak [37] reported that milk oligosaccharides have antioxidant capacity and they suggested detailed investigations to know their medicinal and functional value. Supplementation of ice cream with olein fraction of chia (*Salvia hispanica* L.) improved the free radical scavenging activity of ice cream [38].

### DPPH free radical scavenging activity

Results of DPPH free radical scavenging activity of raw, pasteurized and boiled milk at 0, 3 and 6 days of storage are described in Table 2. DPPH free radical scavenging activity of buffalo milk was higher than cow milk. Pasteurization and boiling did not have any significant effect on DPPH free radical scavenging of both cow and buffalo milk. DPPH free radical scavenging activity of raw, pasteurized and boiled cow milk was 24.3, 23.8 and 23.6%, respectively, whereas, DPPH free radical scavenging activity of buffalo milk was 31.8, 31.5 and 30.4%, respectively. Storage period up to 3 days was non-significant, DPPH assay after 6 days of storage period indicated significant decline in antioxidant activity of raw, pasteurized, boiled cow and buffalo milk. DPPH is one of the most commonly used assay for the determination of antioxidant capacity of dairy products. [39] Used DPPH free radical scavenging assay for the determination of antioxidant capacity of milk. DPPH free radical scavenging activity of yoghurt, acidophilus milk, butter milk and flavored fermented were compared, of the said dairy products, yoghurt revealed the highest antioxidant activity [40]. DPPH free radical scavenging activity of milk and goat milk kefir were compared and kefir revealed stronger antioxidant activity [11].

### Antioxidant Activity in Linoleic Acid (AALA).

Results of free radical scavenging activity in linoleic acid of raw, pasteurized and boiled milk at 0, 3 and 6 days of storage are presented in Table 2. AALA of buffalo milk was more than cow milk. AALA of buffalo and cow milk were 11.7% and 17.4%, respectively. Pasteurization and boiling did not have any effect on AALA in both cow and buffalo milk. AALA of raw, pasteurized and boiled cow milk was 11.7%, 11.3% and 11.1%, respectively. AALA of raw, pasteurized and boiled buffalo milk was 17.4%, 17.1% and 16.8%, respectively. AALA of raw, pasteurized and boiled milk samples up to 3 days of storage period was not different from fresh samples (0 day). AALA of 6 days stored samples in refrigeration was considerably different from fresh samples in raw, pasteurized and boiled milk. AALA of dairy products has been reported in literature. Nadeem et al. [41] reported that supplementation of whey butter with almond peel extract improved the AALA. Supplementation of ice cream with sugarcane juice enhanced the AALA [38]. Fortification of Gouda cheese with mango kernel oil increased the antioxidant capacity of Gouda cheese [42]. Addition of interesterified *Moringa oleifera* oil in ice cream increased the antioxidant capacity [43]. Antioxidant capacity of probiotic yoghurt containing *lactobacillus pentacoccus* was higher than substrate milk [12]. Cow milk has antioxidant capacity, while antioxidant capacity of buffalo milk is not previously studied. In this pioneer study, detailed investigation has been done on antioxidant capacity of raw and thermally processed cow and buffalo milk.

### Effect of pasteurization and boiling on vitamin content of cow and buffalo milk

Milk has two distinct antioxidant systems, fat soluble and water soluble antioxidant system. Fat soluble antioxidant system is mainly comprised of vitamin A and E and water soluble antioxidant is comprised of ascorbic acid and minerals. In current investigation, transition in concentration of vitamins and minerals of cow and buffalo as a function of pasteurization and boiling was used as an indication of change of antioxidant capacity at different stages of storage period. Results regarding influence of pasteurization and boiling on vitamins and minerals content of cow and buffalo milk are presented in Table 3. Amount of vitamin C in raw, pasteurized and boiled cow milk was 0.52, 0.33 and 0.09 mg/100 g, respectively while Vitamin C in raw, pasteurized and boiled buffalo milk was 0.68, 0.39 and 0.26 mg/100 g, respectively. Loss of vitamin C in pasteurization of cow and buffalo milk was 40% and 42%, respectively. The loss of vitamin C in boiling of cow and buffalo milk was 82% and 61%, respectively. Concentration of vitamin A and E in pasteurized cow and buffalo milk was not significantly different from raw milk samples of cow and buffalo. Storage period had a pronounced effect on concentration of vitamin A, E and C. Storage period up to 3 days was non-significant. Vitamin A and E content of boiled cow and buffalo milk samples were significantly less than raw milk samples. Concentration of vitamin A and E and raw and pasteurized milk was not different [44]. Michlova et al. [45] described that concentration of vitamin A during thermal processing and storage period decreased. Saffert et al. [46] studied the effect of UHT treatment and storage period on vitamin A content and UHT treatment and storage period considerably affected vitamin C. All the determination frequencies revealed the decreasing trend during the 12 weeks of storage period. Vitamin E is a powerful antioxidant than vitamin A, concentration of vitamin E also decreased during thermal processing and storage of milk [47]. Effect of heat treatment on vitamins of milk is extensively studied however, little information is available regarding the transition of antioxidant capacity of milk with respect to alteration in vitamin A, E and C. Total antioxidant capacity of cow and buffalo milk were strongly correlated with vitamin A at 0, 3 and 6 days of storage period. Results of selenium and zinc content of raw, pasteurized and boiled cow milk are presented in Table 3. Selenium and zinc contents of buffalo milk were greater than cow milk. Selenium and zinc content of both cow and buffalo milk were not influenced by pasteurization, boiling and storage period of 6 days. Selenium is a trace mineral that is essential, recommended dietary allowance for selenium is 50 μg/day [48]. Antioxidant capacity of selenium is reported in literature, selenium intake reduced the risk of heart disease, cystic fibrosis and cancer [49]. Zn is one of the most significant water soluble antioxidant of milk and essential trace mineral, recommended dietary allowance of Zn is 15 mg/day and use of selenium for the fortification of Turkish white cheese has been reported [50].

**Table 3:** Vitamin and mineral content of raw, pasteurized and boiled cow and buffalo milk

Parameter	Days	Cow Milk			Buffalo Milk		
		Raw Milk	Pasteurized	Boil	Raw Milk	Pasteurize	Boil
Vitamin C mg/100 g	0	0.52 ± 0.03^b^	0.31 ± 0.06^d^	0.09 ± 0.01^g^	0.68 ± 0.07^a^	0.39 ± 0.04^c^	0.26 ± 0.05^e^
	3	0.41 ± 0.02^c^	0.27 ± 0.01^e^	0.07 ± 0.02^g^	0.53 ± 0.09^b^	0.33 ± 0.02^d^	0.23 ± 0.03^e^
	6	0.30 ± 0.05^d^	0.19 ± 0.02^f^	0.03 ± 0.01^h^	0.38 ± 0.10^c^	0.21 ± 0.06^f^	0.11 ± 0.01^g^
Vitamin A μg/100 g	0	62.8 ± 1.57^c^	58.9 ± 1.36^cd^	51.2 ± 0.76^d^	131.9 ± 2.45^a^	129.7 ± 1.17^a^	128.8 ± 1.31^a^
	3	64.5 ± 2.51^c^	55.7 ± 2.61^cd^	49.71 ± 0.95^d^	128.9 ± 3.67^a^	127.8 ± 1.42^a^	128.5 ± 1.72^a^
	6	47.2 ± 0.88^d^	37.5 ± 0.53^e^	28.44 ± 1.08^f^	105.73 ± 1.22^b^	106.58 ± 1.64^b^	103.2 ± 0.98^b^
αTocopherol mg/100 g	0	0.19 ± 0.01^c^	0.17 ± 0.02^c^	0.15 ± 0.01^c^	1.27 ± 0.15^a^	1.21 ± 0.13^a^	1.19 ± 0.08^a^
	3	0.16 ± 0.03^c^	0.14 ± 0.01^c^	0.12 ± 0.02^c^	1.23 ± 0.09^a^	1.16 ± 0.03^a^	1.15 ± 0.02^a^
	6	0.09 ± 0.04^d^	0.08 ± 0.03^d^	0.06 ± 0.01^d^	0.97 ± 0.05^b^	0.95 ± 0.14^b^	0.92 ± 0.07^b^
Selenium μg/100 g	0	0.92 ± 0.13^b^	0.94 ± 0.16^b^	0.91 ± 0.11^b^	6.22 ± 0.10^a^	6.25 ± 0.32^a^	6.18 ± 0.32^a^
	3	0.91 ± 0.08^b^	0.88 ± 0.05^b^	0.88 ± 0.03^b^	6.12 ± 0.13^a^	6.20 ± 0.19^a^	6.14 ± 0.03^a^
	6	0.88 ± 0.15^b^	0.84 ± 0.04^b^	0.86 ± 0.02^b^	6.11 ± 0.06^a^	6.13 ± 0.07^a^	6.11 ± 0.14^a^
Zinc μg/100 g	0	419.7 ± 4.39^b^	417.3 ± 3.94^b^	412.9 ± 3.89^b^	563.5 ± 7.56^a^	559.3 ± 2.43^a^	554.6 ± 3.97^a^
	3	416.5 ± 5.41^b^	415.6 ± 4.22^b^	408.7 ± 2.55^b^	559.7 ± 11.2^a^	554.4 ± 6.82^a^	549.3 ± 7.21^a^
	6	411.8 ± 7.49^b^	413.4 ± 9.27^b^	403.5 ± 6.77^b^	552.6 ± 9.73^a^	550.2 ± 8.52^a^	542.1 ± 2.88^a^

Within the rows and columns of a parameter, means denoted by a different letter are statistically different (*P* < 0.05)

### Fatty acid profile of cow and buffalo milk

Results regarding fatty acid profile of raw, pasteurized and boiled cow and buffalo milk are given in Table 4. Fatty acid profiles of raw cow milk were different from raw buffalo milk. Concentration of short-chain fatty acids (C_4:0_ to C_10:0_) in raw cow and buffalo milk was 10.98% and 15.59%, respectively. Medium-chain fatty acids (C_12:0_ to C_16:0_) in raw cow and buffalo milk were 45.09% and 41.09%, respectively. Long-chain unsaturated fatty acids (C_18:1_ to C_18:3_) in raw cow and buffalo milk were 23.64% and 30.66%, respectively. Fatty acid profile of buffalo milk is different from cow milk [51]. Concentration of oleic acid in buffalo milk was 27.62%. C_18:1_ content in buffalo milk was 29.47% [52]. Nadeem et al. [29] reported that concentration of C_18:1_ and C_18:2_ in cow milk were 23.5% and 1.37%, respectively. Pasteurization and boiling increased the concentration of short-chain fatty acids in both cow and buffalo milk. In pasteurized cow and buffalo milk, concentration of short-chain fatty acids was 12.35% and 15.11%, respectively. In boiled cow and buffalo milk, concentration of short-chain fatty acids was 14.43% and 17.29%, respectively. As a function of heat treatment, long-chain fatty acids may be converted into short-chain and medium-chain fatty acids [53]. Wang et al. [54] reported that concentration of short-chain and medium chain fatty acids increased after pasteurization of goat milk. Both cow and buffalo milk contains reasonable amount of C_18:1_, unsaturated fatty acids play an important in human health. C_18:1_. Fats rich in mono unsaturated fatty acids are getting a great deal of attention for having healthful properties and better oxidative stability. Monounsaturated fatty acids possess the capability of reducing blood cholesterol. C_18:2_ and C_18:3_ can decrease the serum cholesterol and essential fatty acids having major role in childhood development [55]. Fatty acid profile of raw, pasteurized and boiled cow and buffalo milk was also influenced by the storage period. The storage period up to 3 days was non-significant (*p* > 0.05), after this period, concentration of saturated and unsaturated fatty acids significantly decreased from the initial vale. Determination of fatty acid profile provides useful indication of oxidation status of milk and dairy products during processing and storage conditions, because accelerated oxidation conditions are usually not used for milk and dairy products. Fatty acid profile of processed and unprocessed goat milk was not similar to each other [56].

**Table 4:** Effect of pasteurization and boiling on fatty acid profile of cow and buffalo milk (g/100 g)

Fatty Acid	Day	Cow Milk			Buffalo Milk		
		Raw	Pasteurized	Boil	Raw	Pasteurized	Boil
C_4:0_	0	3.75 ± 0.04^b^	3.82 ± 0.03^b^	3.94 ± 0.05^a^	3.98 ± 0.09^a^	4.11 ± 0.08^a^	4.33 ± 0.09^a^
	6	3.58 ± 0.13^c^	3.79 ± 0.02^b^	3.81 ± 0.07^b^	3.88 ± 0.13^b^	3.95 ± 0.11^b^	4.15 ± 0.07^b^
C_6:0_	0	2.28 ± 0.07^j^	2.87 ± 0.16^f^	3.35 ± 0.17^d^	2.41 ± 0.13^h^	3.96 ± 0.21^b^	4.19 ± 0.14^a^
	6	2.21 ± 0.08^k^	2.71 ± 0.03^g^	3.26 ± 0.08^e^	2.32 ± 0.05^i^	3.61 ± 0.09^c^	4.03 ± 0.06^b^
C_8:0_	0	1.53 ± 0.02^i^	1.68 ± 0.08^h^	1.92 ± 0.16^g^	2.53 ± 0.17^e^	2.81 ± 0.19^c^	3.35 ± 0.22^a^
	6	1.71 ± 0.05^h^	1.91 ± 0.03^g^	2.56 ± 0.04^e^	2.39 ± 0.29^f^	2.71 ± 0.12^d^	3.19 ± 0.16^b^
C_10:0_	0	3.42 ± 0.03^g^	3.98 ± 0.05^h^	5.22 ± 0.43^b^	3.67 ± 0.11^f^	4.23 ± 0.06^d^	5.42 ± 0.07^a^
	6	3.32 ± 0.31^h^	3.73 ± 0.15^i^	4.89 ± 0.27^c^	3.48 ± 0.13^g^	3.97 ± 0.03^e^	5.27 ± 0.02^b^
C_12:0_	0	4.25 ± 0.06^g^	5.34 ± 0.20^e^	6.55 ± 0.18^b^	3.15 ± 0.19^i^	5.33 ± 0.14^e^	6.86 ± 0.21^a^
	6	4.11 ± 0.26^h^	5.15 ± 0.38^f^	6.38 ± 0.41^c^	3.03 ± 0.04^j^	5.11 ± 0.05^f^	5.66 ± 0.07^d^
C_14:0_	0	11.68 ± 0.29^i^	12.87 ± 0.49^e^	14.59 ± 0.67^a^	10.72 ± 0.08^k^	12.19 ± 0.53^f^	13.45 ± 0.72^c^
	6	11.15 ± 0.62^j^	11.12 ± 0.36^h^	14.11 ± 0.43^b^	10.13 ± 0.92^l^	11.66 ± 0.83^g^	12.47 ± 0.27^d^
C_16:0_	0	29.16 ± 0.67^g^	31.45 ± 1.24^d^	34.61 ± 1.65^a^	27.88 ± 0.94^j^	29.82 ± 1.44^f^	32.95 ± 1.87^b^
	6	28.72 ± 0.99^h^	30.83 ± 1.15^e^	32.76 ± 1.38^b^	25.41 ± 1.55^i^	27.55 ± 1.67^j^	31.44 ± 0.94^c^
C_18:0_	0	9.35 ± 0.38^d^	9.21 ± 0.73^d^	9.13 ± 0.52^e^	12.77 ± 0.33^a^	11.24 ± 0.53^c^	9.37 ± 0.19^d^
	6	9.17 ± 0.46^e^	8.83 ± 0.42^f^	8.67 ± 0.38^g^	12.05 ± 0.66^b^	10.41 ± 0.45^c^	8.62 ± 0.32^g^
C_18:1_	0	21.18 ± 0.97^f^	19.23 ± 1.77^h^	18.66 ± 0.54^i^	27.65 ± 0.73^a^	26.39 ± 1.14^b^	25.12 ± 1.19^c^
	6	20.06 ± 0.97^g^	18.34 ± 0.85^i^	17.92 ± 0.46^j^	26.18 ± 0.65^b^	24.59 ± 1.35^d^	23.44 ± 0.33^e^
C_18:2_	0	1.98 ± 0.05^d^	1.72 ± 0.03^e^	1.37 ± 0.21^h^	2.37 ± 0.16^a^	2.21 ± 0.13^b^	1.91 ± 0.16^d^
	6	1.56 ± 0.08^g^	1.62 ± 0.11^f^	1.19 ± 0.06^i^	2.12 ± 0.13^c^	1.92 ± 0.03^d^	1.51 ± 0.13^g^
C_18:3_	0	0.48 ± 0.02^b^	0.35 ± 0.04^d^	0.22 ± 0.05^e^	0.64 ± 0.01^a^	0.55 ± 0.08^b^	0.43 ± 0.06^c^
	6	0.27 ± 0.03^d^	0.19 ± 0.01^e^	0.13 ± 0.02^f^	0.52 ± 0.03^b^	0.44 ± 0.02^c^	0.31 ± 0.02^d^

Within the rows and columns of a fatty acid, means denoted by a different letter are statistically different (*P* < 0.05)

### Peroxide value

Effects of pasteurization, boiling and storage period on peroxide value of raw, pasteurized and boiled cow and buffalo milk are described in Table 5. Peroxide value of pasteurized and boiled cow and buffalo milk samples was greater than raw milk samples of cow and buffalo. Peroxide value of raw, pasteurized and boiled cow milk was 0.22, 0.27 and 0.39 (MeqO_2_/kg), respectively. Peroxide value of raw, pasteurized and boiled buffalo milk was 0.24, 0.29 and 0.37(MeqO_2_/kg), respectively. Peroxide value of raw, pasteurized and boiled cow and buffalo milk was not significant up to 3 days of refrigeration storage, determination of peroxide value after 6 days of storage period revealed significant difference from fresh and 3 days old samples of both cow and buffalo milk. The significant rise in peroxide value of raw and thermally processed cow and buffalo milk may be due to the decrease in antioxidant capacity, results of peroxide value and antioxidant capacity of 6 days stored cow and buffalo milk were in line with each other. The rise in peroxide value of cow milk as a function of pasteurization and boiling is reported in literature. Mashref et al. [57] studied the impact of pasteurization and boiling on peroxide value of cow milk and peroxide value of pasteurized and boiled milk was greater than raw milk. Peroxide value of cow milk increased during the storage period of 6 days [29]. However, the oxidative stability of buffalo milk is not studied in detail.

**Table 5:** Effect of pasteurization and boiling on peroxide value of cow and buffalo milk

Day	Cow Milk			Buffalo Milk		
	Raw	Pasteurized	Boil	Raw	Pasteurized	Boil
0	0.22 ± 0.02^c^	0.27 ± 0.05^c^	0.39 ± 0.07^b^	0.24 ± 0.13^c^	0.29 ± 0.05^c^	0.37 ± 0.12^b^
3	0.25 ± 0.01^c^	0.30 ± 0.06^c^	0.43 ± 0.05^b^	0.26 ± 0.09^c^	0.30 ± 0.08^a^	0.42 ± 0.04^b^
6	0.37 ± 0.04^b^	0.41 ± 0.03^b^	0.48 ± 0.11^a^	0.31 ± 0.06^c^	0.37 ± 0.10^b^	0.51 ± 0.07^a^

Within the rows and columns, means denoted by a different letter are statistically different (*P* < 0.05)

### Sensory evaluation

Effect of pasteurization and storage period of 6 days on colour and flavour of cow and buffalo milk is described in Table 6. Colour of raw buffalo milk was white and raw cow milk was slightly yellowish. Pasteurization and boiling did not have any effect on colour and flavour score of both cow and buffalo milk. Higher colour and flavour score of all the three version of buffalo milk was due to the taste preference of buffalo milk in the sub-continent. Storage period up to 3 days was non-significant in raw, pasteurized and boiled cow and buffalo milk. After 3 days of storage, colour and flavour score was remarkably decreased (*p* < 0.05).

**Table 6:** Effect of pasteurization and boiling on sensory characteristics of cow and buffalo milk

Parameter	Day	Cow Milk			Buffalo Milk		
		Raw	Pasteurized	Boil	Raw	Pasteurized	Boil
Color	0	7.5 ± 0.55^b^	7.4 ± 0.42^b^	7.4 ± 0.09^b^	8.1 ± 0.39^a^	8.0 ± 0.15^a^	8.2 ± 0.27^a^
	3	7.4 ± 0.40^b^	7.3 ± 0.48^b^	7.2 ± 0.26^b^	8.0 ± 0.14^a^	7.9 ± 0.11^a^	8.1 ± 0.22^a^
	6	7.1 ± 0.23^c^	6.8 ± 0.37^d^	6.7 ± 0.30^d^	7.5 ± 0.24^b^	7.4 ± 0.13^b^	7.3 ± 0.19^b^
Flavor	0	Not Determined	7.5 ± 0.40^b^	7.5 ± 0.06^b^	Not Determined	8.2 ± 0.07^a^	8.0 ± 0.19^a^
	3		7.3 ± 0.28^b^	7.4 ± 0.08^b^		8.0 ± 0.17^a^	7.8 ± 0.11^a^
	6		6.9 ± 0.18^c^	7.0 ± 0.08^c^		7.5 ± 0.38^b^	7.6 ± 0.26^b^

Within the rows and columns, means denoted by a different letter are statistically different (*P* < 0.05)

## Conclusions

The results of the present study concluded that Antioxidant capacity of buffalo milk was more than cow milk while pasteurization and boiling did not any effect on antioxidant capacity, vitamin E, A, zinc, selenium and sensory characteristics of cow and buffalo milk. Antioxidant capacity of buffalo and cow milk decreased after 3 days of refrigerated storage. These results suggest that buffalo milk has more health prospects than cow milk.
